# Prof. Erichsen and American Surgery

**Published:** 1875-01

**Authors:** 


### MISCELLANEOUS.

### Prof. Erichsen on American Surgery.

We copy from correspondence of the *Pacific Medical and Surgical Journal* the following remarks by Prof. Erichsen on the Medical Profession in America:

I was the subject of flattering attention far beyond my expectation, but this I referred rather to the fact of being considered a representative of English surgery than to any personal merit as an individual. I was pleased to observe the high social status occupied by the profession in America, which seemed, indeed, to be above that of the other learned professions there; and this arises in part from the fact that the legal profession there occupies a position at least uncertain, if not equivocal, and, as regards the clergy, their position is less exalted from the non-existence of the ecclesiastical hierarchy found in our own country. American surgery is eminently practical and progressive; not restrained by old doctrines, it is ever on the alert to adopt whatever may seem to be good in the suggestions of the profession abroad.

From a very extended range of observation—New York, Philadelphia, Boston, Washington and Chicago—I am free to acknowledge that the American physicians are the best read professional men I have ever met with. I was frequently surprised at their range of general knowledge, and would offer as an explanation that they are wedded neither to the old or the new, since in their intellectual culture these two elements are fused together. I cannot do better than to make use off an old classic quotation: *Cœlum, non animum, mutant, qui trans mare currunt*, which, anglicized, is: They change their sky, not their character, who cross the seas; for I have found the people much like Englishmen; they have the same energy, the same determination of purpose, and, as physicians, they look on patients as men, not as philosophical objects of inquiry. Though progressive and fond of the new, there is still found with them a love of the traditional. I found that they had drank from the same fountains whence we draw our inspiration, viz., from those of British source; for example, in Philadelphia, at the Pennsylvania Hospital, my attention was attracted to a fine portrait which, at first sight, I supposed might be some American medical or surgical celebrity, but, approaching, I found it to be that of our own Sir Astley Cooper.

I found English medical literature to be exceedingly well-known in the United States; that many medical works from our country are republished there, and especially, that the *London Lancet* was reprinted by them and had quite as general a circulation among them as among us.

The course of medical education in the United States is, in many respects, similar to our own, though the time devoted to study is somewhat shorter; their curriculum consists of three years, whilst ours is four years. I, however, regretted to find that in one respect their system has a great fault, viz., the absence of a matriculation examination.

I was astounded at the number of students present at some institutions; for example, at an introductory lecture which I attended in Philadelphia, there were six hundred students present; and at Bellevue Hospital, in New York City, where it was announced that I was to operate, (which, however, I declined to do) there were present nearly a thousand students.

It results, from the great number of young men attending the medical schools, that it is impossible for the class to be brought to the bedside during clinical teaching, and since the students cannot be brought to the patient, they have remedied the matter by bringing the patient to the students, and, certainly in surgical cases, this seems to answer quite as well as our plan of bringing the class to the bedside.

Dissecting material is abundant and costs but little, since the bodies of many dying in the public hospitals are used for that purpose. [The allusion to the cheapness of material caused his class to cheer him.]

American hospitals are almost all supported by voluntary contributions; but from my observations, I think that these benevolent institutions are far less abused by improper subjects gaining admission to them, than is the case with us; for here, I am sorry to say, that too often charity covers a multitude of evils. The Americans spend their money very freely, both for their pleasures and in contributions to public charities.

I found two classes of hospitals; they might be designated as the old and the new. The old I may name the pre-sanitary, being entirely behind hand in sanitary arrangements. During their late war the Americans learned a hard lesson, viz., that aggregation of patients caused fearful mortality, whilst those quartered in tents and barracks, thereby having much more room, did far better; these facts were quickly noted and taken advantage of; thence sprung the pavilion hospital. I will however say, that with us the pavilion system would be exceedingly expensive on account of its covering so much ground. In New York City the pavilion system or barracks is on the eve of construction at Bellevue, and also for the Hospital for Women. The most perfect hospital I saw in New York was the Roosevelt, which consists of three parts, viz., a central one for general administrative purposes, and lateral wings for hospital purposes. At this hospital I saw a ward fitted up for surgical cases, where all the details of modern hygiene are so carefully carried out, that since the construction of the building, there has occurred but one case of pyemia, and even this was of doubtful character.

In the United States the different religious sects are in the habit of building hospitals; for instance, Catholic hospitals, Presbyterian hospitals, Episcopal hospitals, etc. (Laughter.)

One great element predominant in the American character is discontent, and this leads them to try new methods; in this way we can account for their constant progress. For example, at Washington City, Dr. Billings is having a new hospital constructed which will combine all late improvements; all the cooking is to be done above, being so isolated that the air cannot pass thence to the wards; besides, through the entire building the air is to be kept in circulation by means of a huge fan-like machine propelled by steam. The arrangements in the American hospitals for extinguishing fire are of a most excellent character and superior to anything which we have.

In their surgical cases I observed that the wounds are dressed very lightly, while, at the same time, special attention is given to drainage. They pay less attention to the antiseptic plan than we do, but this arises from the fact that it is unnecessary, since their hospitals are maintained so nearly free from all septic influences. For, it is my opinion, were hospitals kept clean, antiseptics would scarcely be needed. If I were to draw a comparison, I should say that in these matters we begin at the wrong end, the Americans at the right one, since we aim at eliminating morbid influences after allowing them to enter, while the main endeavor of the American surgeon is to prevent their entrance.

It is well you should know that anesthetics were discovered by Americans, having first been used at the Massachusetts General Hospital.

There are fewer museums there than here, though they have a few remarkable ones; among these I may mention Hyrt's collection of anatomical preparations, which has been purchased by one of the medical schools. Also, the Army Medical Museum at Washington deserves special notice. I must likewise mention the surgical works that have been issued by the Medical Bureau of the U. S. Army; in material and in illustration they excel anything ever published by any government. So unique and wonderful are many of the specimens in the Army Medical Museum illustrating wounds of the cranium, as also wounds inflicted by arrows, that to see them is almost worth a journey to the United States.

In the excision of bones, and especially of joints, the American surgeons have made a progress of which they may be truly proud.

I shall refrain from especially naming the leading surgical celebrities of the United States, since, by so doing, I might make some invidious distinctions, and the necessity of doing so is less from the names of many of them being quite as familiar to you as those of English surgeons. In conclusion, if at the end of your student's course you be not satisfied *in verba magistri jurare*, I advise you, instead of as heretofore visiting France and Germany, to visit the United States.

